# Preoperative early-stage lung cancer patients and local brain area changes: a cross-sectional observational descriptive study

**DOI:** 10.3389/fpsyg.2024.1417668

**Published:** 2024-08-14

**Authors:** Pei-Pei Yuan, Xu-Yun Hua

**Affiliations:** ^1^Department of Nursing, Shuguang Hospital, Shanghai University of Traditional Chinese Medicine, Shanghai, China; ^2^Department of Orthopedics, Shuguang Hospital, Shanghai University of Traditional Chinese Medicine, Shanghai, China

**Keywords:** early-stage lung cancer, functional magnetic resonance imaging, neuroplasticity, emotion, negative emotion, amplitude of low-frequency

## Abstract

**Introduction:**

Lung cancer is a major global health concern. Patients undergo a substantial process of emotional transformation following a lung cancer diagnosis, during which subtle changes in brain function and/or structure may occur. As such, the present study aimed to investigate the neuroplastic changes induced by negative emotions in patients with early-stage lung cancer.

**Methods:**

This cross-sectional study recruited 35 patients with early-stage lung cancer and 33 age- and sex-matched healthy control patients. All participants completed the Hamilton Anxiety Rating Scale (HAMA), Hamilton Depression Rating Scale (HAMD), and functional magnetic resonance imaging (fMRI). Amplitude of low-frequency fluctuations (ALFF) and regional homogeneity (ReHo) were used as the fMRI indices. Correlations between the clinical assessments and ALFF and ReHo values were calculated.

**Results:**

Our analysis revealed no significant differences in HAMD and HAMA scores between patients and control patients (*p* > 0.05). However, significant alterations in ALFF and ReHo were observed in multiple brain regions in patients with early-stage lung cancer compared to healthy controls (*P*_FalseDiscoveryRate_ < 0.05). Specifically, ALFF values were decreased in the right postcentral gyrus, calcarine, and left middle cingulate, while ReHo values increased in the right angular gyrus and decreased in the bilateral postcentral gyrus, insula, left calcarine, putamen, superior temporal gyrus, middle cingulate, and right Rolandic gyrus. The HAMD score was significantly correlated with the ALFF value in the right postcentral gyrus (*P* = 0.007).

**Conclusion:**

This study provides valuable insights into the adaptive responses of the brain following the early diagnosis of lung cancer, revealing potential disturbances in negative emotional processing. Harnessing neuroplasticity may open new avenues for the establishment of personalized treatment strategies and targeted interventions to support the emotional and mental health of patients with lung cancer.

## 1 Introduction

Lung cancer is the third most common cancer and the leading cause of cancer-related death in China (Yang et al., 2018). Relevant studies have reported that the 5-year survival rate of patients with lung cancer ranges from 4 to 17%, depending on the stage and regional variations (Kessler et al., 2023). The stage at diagnosis has a significant impact on treatment strategy and prognosis (Hirsch et al., 2017). With the widespread use of low-dose computed tomography and increased awareness of medical care among the Chinese population, routine medical checkups have improved the rates of early detection and treatment of lung cancer (Siegel et al., 2020). Compared to advanced cancer, patients diagnosed with early-stage cancer are able to receive timely radical treatment and, therefore, have a better prognosis (Zeng et al., 2021).

Nevertheless, the negative emotions associated with cancer diagnosis, such as overwhelming fear, worry, anger, sadness, depression, stress, and anxiety, are intense. The mental health issues triggered by cancer can have unmanageable negative effects on the lives of patients and their families. Studies have reported that the rates of clinically significant disorders such as depression, failure to adjust, alcohol use disorder, and insomnia rapidly increased following diagnosis (Hopwood and Stephens, 2000). Cancer patients require substantial psychological support to manage their diagnosis and treatment (Schulze et al., 2014). Therefore, changes in neuroplasticity that occur in patients during this period after cancer diagnosis are very important. Neuroplasticity, defined as the ability of the brain to grow and evolve in response to life experiences, encompasses the extraordinary capacity of the brain to adapt and reorganize in response to both internal and external stimuli (Merzenich et al., 2013). Intriguingly, they may have played an unexpected role in the disease evolution. As lung cancer progresses, even before clinical symptoms become apparent, subtle changes in brain function and/or structure may occur, particularly when influenced by emotions. These adaptive plasticity mechanisms in early-stage lung cancer may explain the mental disorders associated with symptom manifestations.

Functional magnetic resonance imaging (fMRI) has helped further explain the adaptive response of the brain to negative emotions related to lung cancer diagnosis. Using available techniques, researchers can directly observe and quantify the changes in neuroplasticity (Liu et al., 2022). Numerous studies have examined functional brain changes specifically associated with chemotherapy in patients with lung cancer. In another study, You et al. (2020) reported a decrease in functional connectivity (FC) values in the frontal and parietal gyri after chemotherapy and a significant decrease in FC values in the default mode network. Liu et al. (2022) have suggested that alterations in the local and global efficiency of the white matter network in the brain are associated with cancer-induced cognitive and affective deficits in patients with non-small cell lung cancer. Another study showed that chemotherapeutic drugs can produce a wide range of effects in the default mode network and executive control network (Hu et al., 2022). Previous studies have clarified the altered central plasticity during lung cancer treatment. However, few studies have focused on the neuroplasticity of negative emotions in patients with early-stage lung cancer.

The amplitude of low-frequency fluctuations (ALFF) and regional homogeneity (ReHo) will be used as fMRI metrics to provide valuable information for understanding the adaptive responses of the regional brain. ALFF is a stable characteristic that facilitates the calculation of the amplitude of each voxel in a localized brain region over a frequency range of 0.01–0.08 Hz. This metric can further be used to quantify the local properties of fMRI signals (Zhu et al., 2021). ReHo is another measure based on the Kendall coefficient of consistency (KCC) used to assess the similarity of the time series of a specific voxel to the time series of its nearest neighboring voxel (Zhu et al., 2021). Therefore, we used both of these metrics to objectively assess spontaneous regional brain activity and to investigate the effects of negative emotions on neuroplasticity in patients with early-stage lung cancer.

## 2 Method

### 2.1 Participants

This study employed a cross-sectional design. A total of 35 patients with early-stage lung cancer, diagnosed based on computed tomography (CT) results by the same thoracic surgeon and pathologist, were recruited from Shanghai Yueyang Hospital between January 2021 and December 2022. The inclusion criteria were as follows: age between 18 and 80 years, no sex limitation, and lung staging meeting the classification criteria of the American Joint Committee on Cancer (AJCC) (Qiu et al., 2011). The diagnostic criteria for patients with lung cancer were based on the identification of ground-glass nodules with a diameter > 8 mm after the first CT scan, with no reduction or disappearance of the nodule in the follow-up CT scan 3 months later, confirmed by pathological manifestations, and with no obvious clinical symptoms. The exclusion criteria were as follows: severe cardiovascular disease, hepatic or renal disease, corticosteroid therapy, pregnancy or lactation, other malignant diseases, severe infections or hematologic disorders, and neuropsychiatric disorders. Moreover, 33 age-gender-matched healthy controls were recruited for this study. Healthy participants were carefully screened to ensure that they did not have a history of lung cancer or any other significant medical conditions.

This study was approved by the Ethics Review Board of Yueyang Hospital of Integrated Chinese and Western Medicine and was conducted in accordance with the guidelines of the Declaration of Helsinki (No. 2021-027). Each participant was fully informed of the study and signed a consent form prior to the study.

### 2.2 Clinical data assessments

General information about the participants in both groups was collected, including age, sex, and smoking history. The primary outcome was to investigate the symptoms of anxiety. The Hamilton Anxiety Rating Scale (HAMA) was adopted in the current study (Beneke, 1987). This scale comprises 14 items, each defined by a series of symptoms: mental anxiety (mental irritability and psychological distress) and somatic anxiety (physical discomfort associated with anxiety). Each item was rated on a scale ranging from 0 (absent) to 4 (severe), with a total score range of 0–56, where a score of < 17 indicated mild, 18–24 indicates mild to moderate, and 25–30 indicates moderate to severe.

We measured depression using the Hamilton Depression Rating Scale (HAMD) and interviewed participants for symptoms of depression over the past week, focusing on the melancholic and somatic symptoms of depression. The HAMD comprises four items designed to typify depression, with scores of 0–7 generally considered to be in the normal range (or in clinical remission), whereas a score of 20 or more (indicating at least moderate severity) (Lee et al., 2019).

### 2.3 fMRI data acquisition

The patients underwent fMRI after the second CT scan. All fMRI data were acquired using a Magnetom Trio A 3T MR scanner (Siemens AG, Erlangen, Germany). During the resting-state fMRI session, the participants were instructed to relax with their eyes closed and keep their heads still. Functional images were subsequently acquired in the same slice orientation with an EPI (gradient recalled echo, echo-planar imaging) sequence (TR/TE = 3,000/30 ms, FOV = 24.0 × 24.0 cm^2^, flip angle = 90°, matrix = 64 × 64, slice thickness = 3.0 mm, slice gap = 0.4 mm, 43 slices, number of acquisitions = 240, acquisition voxel size = 3.0 × 3.0 × 3.0 mm).

### 2.4 fMRI data preprocessing

The RESTplus (http://restfmri.net/forum/RESTplusV1.2) was used to perform data preprocessing, including removing the first 10 volumes, correcting slicing time, realigning head motion correction, normalizing to EPI standard templates, and resampling to 3.0 × 3.0 × 3.0 mm^3^.

The data were smoothed using a 6-mm FWHM isotropic Gaussian kernel. After checking, excluding the image of movements that exceeded 2.0 mm or rotations that exceeded 2.0° (Jia et al., 2020).

### 2.5 Statistical analysis

All statistical analyses were conducted using IBM SPSS (IBM for Windows version). Continuous variables were summarized as means ± standard deviations (independent sample *t*-tests) and presented as frequencies and percentages (χ2 test). Data were tested for normality using the Shapiro-Wilk test before applying independent sample *t*-tests. All statistical tests were two-tailed. Statistical significance was set at a *p*-value of < 0.05. Differences in ALFF and ReHo values between the two groups were analyzed using a two-sample *t*-test based on RESTplus, and the statistical significance was set at *p* < 0.05_(FalseDiscoveryRate(FDR)correct)_. The correlation between the clinical assessments and ALFF and ReHo values was calculated using Pearson's correlation analysis. Statistical significance was set at a *p*-value of < 0.05.

## 3 Results

### 3.1 Patient characteristics

The study cohort comprised 35 patients with early-stage lung cancer and 33 healthy patients. No significant differences in sex, age, smoking years, HAMD, or HAMA were found between the patients and control patients (*p* > 0.05). The demographic and clinical data of both groups are listed in Table 1.

**Table 1:** Characteristics of the recruited patients.

**Characteristics**	**Group**		***p*-value**
	**Early-stage lung cancer (*n* = 35)**	**Healthy control (*n* = 33)**	
Age (years)	65.5 ± 10.0	63.1 ± 8.6	0.303
Gender (male/female)	23/12	23/10	0.927
Smoke History	25.71%	21.21%	0.880
HAMD	12.4 ± 3.1	13.6 ± 4.0	0.167
HAMA	11.1 ± 3.7	12.1 ± 4.0	0.307

### 3.2 Group comparisons of ALFF value

**Figure 1:**
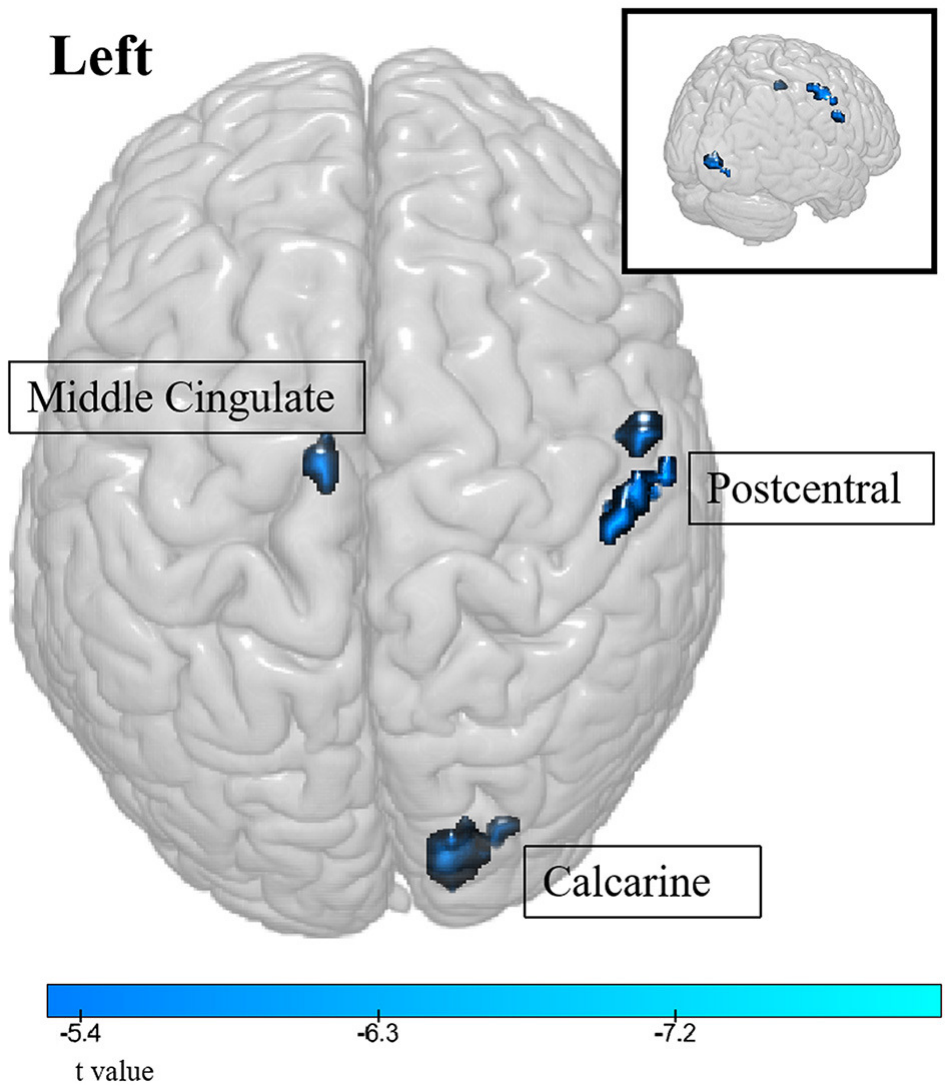
The differences in ALFF between the cancer group and healthy group. Cool color indicates that the ALFF value in the cancer group was lower than in the healthy group.

Compared to healthy controls, lung cancer patients showed significant differences in multiple brain regions (Table 2; Figure 1). The ALFF decreased in the right postcentral gyrus, calcarine, and left middle cingulate (*P*_FDR_ < 0.05).

**Table 2:** Comparison of amplitude of low-frequency fluctuations (ALFF) between the cancer group and the healthy group.

**Contrast name**	**Region label**	**Extent**	***t*-value**	**MNI coordinates**		
				***x***	***y***	***z***
Negative	Right postcentral	38	−7.4279	54	−6	30
	Right calcarine	35	−7.393	15	−90	0
	Left middle cingulate	10	−7.3435	−9	−12	42

### 3.3 Group comparisons of Reho value

**Figure 2:**
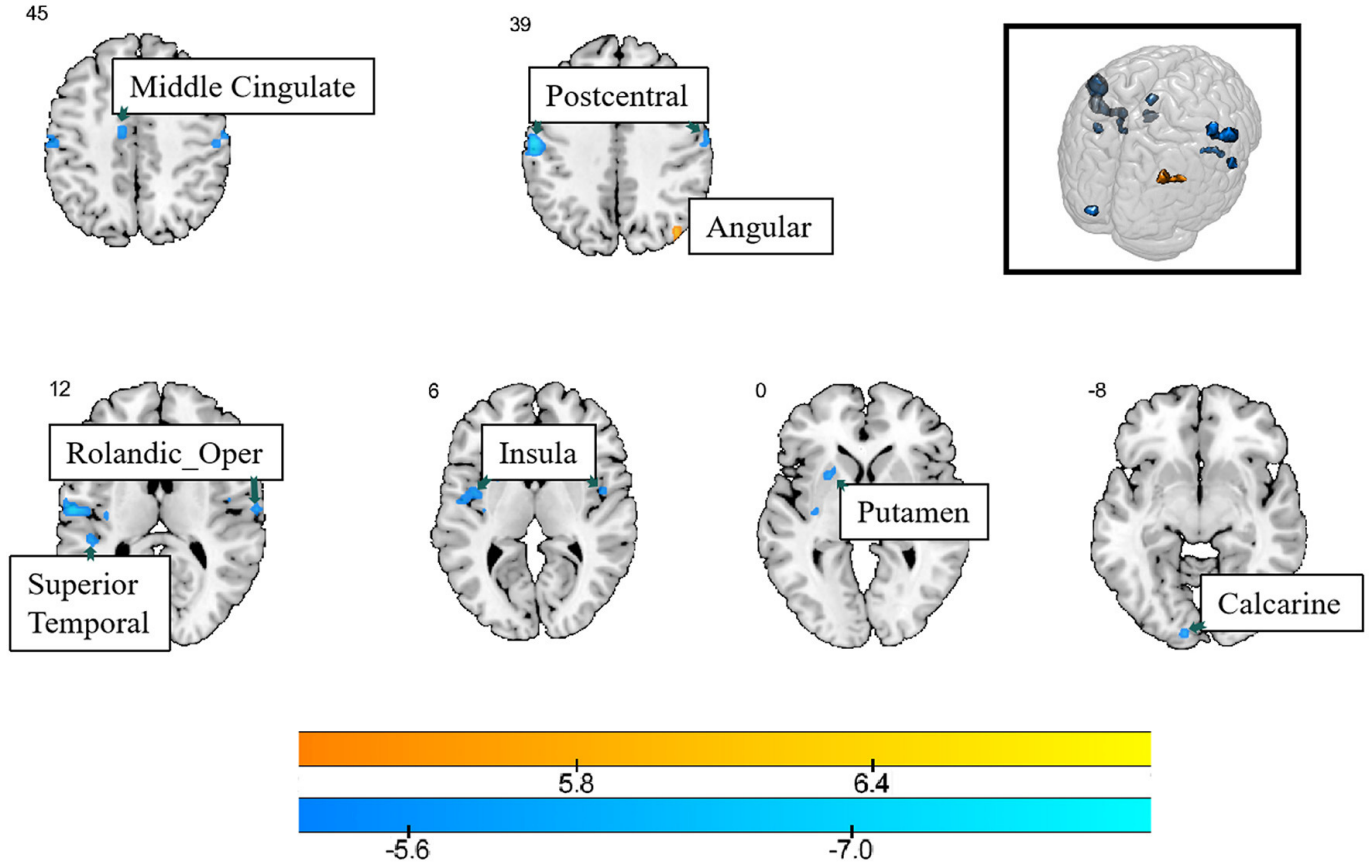
The differences in ReHo between the cancer group and healthy group. Cool color indicates that the ReHo value in the cancer group was lower than in the healthy group, while hot color indicates vice versa.

Compared with healthy controls, lung cancer patients showed increased Reho values in the right angular gyrus, and several brain regions showed decreased Reho values, such as the bilateral postcentral gyrus, insula, left calcarine, putamen, superior temporal gyrus, middle cingulate, and right Rolandic gyrus (*P*_FDR_ < 0.05) (Table 3; Figure 2).

**Table 3:** Comparison of regional homogeneity (ReHo) between the cancer group and healthy group.

**Contrast name**	**Region label**	**Extent**	***t*-value**	**MNI coordinates**		
				***x***	***y***	***z***
Positive	Right angular	33	6.3406	39	−75	39
Negative	Left postcentral	218	−7.9504	−57	−18	36
	Right Rolandic_Oper	41	−6.4094	63	−12	12
	Left calcarine	13	−6.3288	−9	−96	−6
	Left putamen	20	−6.2447	−24	12	0
	Left superior temporal	14	−6.2281	−45	−33	12
	Right insula	24	−6.1905	45	0	6
	Left middle cingulate	10	−6.1653	−9	−12	45
	Left insula	107	−6.1154	−42	−3	6
	Right postcentral	68	−6.0488	51	−24	54

### 3.4 Correlation analysis

**Figure 3:**
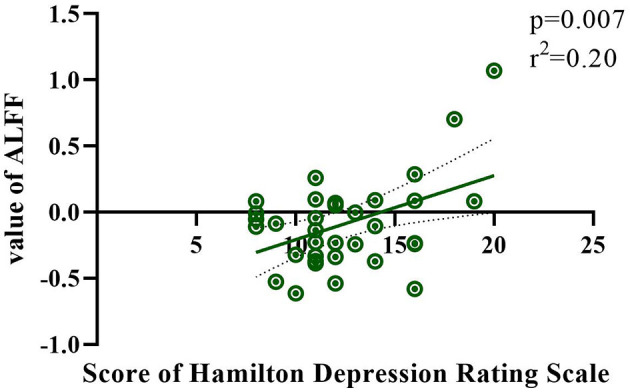
The correlation between the HAMD score and ALFF value in the right postcentral gyrus.

The correlation between HAMD/HAMA and ALFF/ReHo values was calculated for each patient using linear regression methods. The HAMD score was found to be significantly correlated with the ALFF value in the right postcentral gyrus (*r*^2^ = 0.20; *p* =0.007) in Figure 3. No other significant correlations were observed between groups.

## 4 Discussion

This is the first study to investigate the role of negative emotions in neuroplastic changes in patients with early-stage lung cancer. Changes in the level of brain activity in cancer patients showed a downward trend compared with healthy controls. However, unexpectedly, we found no significant differences in HAMD/HAMA scores between the two groups. The observed significant alterations in brain regions of early-stage lung cancer patients may have important implications for understanding the impact of the disease on the brain and its functional consequences and may open new avenues for the future development of targeted interventions and personalized therapeutic strategies.

Our observation of decreased ALFF in the right postcentral gyrus, calcarine, and left middle cingulate in patients with lung cancer suggests a possible disturbance of sensory processing and attentional functions. The postcentral gyrus contains the primary somatosensory cortex and is involved in somatosensory processing; a decrease in its activity may indicate altered sensory perception in lung cancer patients (Roland et al., 1998).

When applying fMRI to monitor brain activity in patients while viewing an emotional movie, Lettieri et al. (2019) found that the right temporoparietal territories, including the postcentral gyrus, encode the polarity, complexity, and intensity of emotional experience. Calcarine plays a crucial role in visual processing, and its decreased activity may be related to changes in visual perception or visual processing deficits in these patients (Chaarani et al., 2022). A study reported that an increase in negative emotions after sleep deprivation significantly changed the functional connectivity between the left thalamus and right calcarine is significantly changed (Li et al., 2021). Additionally, the left middle cingulate, which is a part of the limbic system, has been implicated in emotional processing and pain modulation (Peyron et al., 2019; Rolls, 2019). Generally, reduced activity in this region may be associated with mood disorders and pain in patients with lung cancer, which are commonly reported symptoms of cancer patients (Heneghan and Kesler, 2023).

It is clear that changes in ReHo values occurred primarily in brain regions associated with emotional regulation. The ReHo value only increased in the right angular gyrus. However, more brain regions showed decreased ReHo values, including the bilateral postcentral gyrus, insula, left calcarine, putamen, superior temporal gyrus, middle cingulate, and right Rolandic gyrus. The angular gyrus has been implicated in a variety of cognitive functions, including attention, memory, and visual-spatial processing (Cattaneo et al., 2009). Involvement of the angular gyrus in the default mode network. This increased activity may reflect a compensatory mechanism for coping with cognitive deficits in patients with lung cancer. This result indicates the need for increased cognition-related research. In regions with decreased activity, the ability of the insula to effectively integrate emotional and sensory information may be disturbed, leading to mood disorders in cancer patients (Ebisch et al., 2015). Altered functional integration in the insula may affect the patients' ability to cope with cancer-related emotional challenges. The decreased values in the putamen may indicate a disruption in communication and coordination within the basal ganglia circuitry, which may affect emotional processing and motor control in lung cancer patients (Azqueta-Gavaldon et al., 2020). Alterations in ReHo in the superior temporal and middle cingulate gyri may lead to communication difficulties and mood disorders in lung cancer patients (Maywald et al., 2023). One prior study reported patterns of distribution of gray matter alterations in multiple brain regions for subclinical depression, anxiety, and somatoform symptoms, with depression scores positively correlated with gray matter in the Rolandic operculum, left superior temporal gyrus, and bilateral postcentral gyrus (Besteher et al., 2017). This is consistent with the brain regions altered in the present study and was confirmed by the positive correlation between HAMD and ALFF scores in the right postcentral gyrus. These results suggest that although the patients did not show significant differences in their HAMD/HAMA scores, the brain's functional activity showed subtle changes.

The significant alterations in the brain regions of early-stage lung cancer patients suggest a complex interaction between the disease and the functional organization of the brain. These changes may account for emotional disturbances and psychological symptoms that occur in patients with lung cancer before the onset of significant clinical symptoms. However, surprisingly, we found no significant differences in the HAMD/HAMA scores of the patients in this study compared to healthy controls. These neuroplasticity changes observed in patients with early-stage lung cancer may provide important insights into the adaptive response of the brain to cancer before the onset of obvious clinical symptoms. This study also has several limitations: Numerous patients will choose other hospitals for treatment or surgery after diagnosis. Therefore, we increased the sample size to include follow-up studies. Moreover, assessment scales for cognitive function should be added. In addition, we will conduct an ongoing longitudinal study in patients who have received chemotherapy, surgery, and other follow-up treatments.

In conclusion, we propose that the changes in brain activity observed in this study may underlie the mechanisms associated with early-stage lung cancer that delay symptom onset. In addition, specific patterns of neuroplasticity observed in patients with lung cancer compared to healthy controls have the potential to serve as novel biomarkers for early-stage lung cancer. Understanding the impact of these altered brain regions in early-stage lung cancer is critical for developing targeted interventions to support patients' emotional and mental health. By identifying the specific brain regions involved in lung cancer-related changes, future research could explore innovative therapeutic approaches to address the negative emotional challenges faced by patients with early-stage lung cancer.

## Data availability statement

The original contributions presented in the study are included in the article, further inquiries can be directed to the corresponding author.

## Ethics statement

The studies involving humans were approved by Ethics Review Committee of Yueyang hospital of integrated Chinese and western medicine. The studies were conducted in accordance with the local legislation and institutional requirements. The participants provided their written informed consent to participate in this study.

## Author contributions

P-PY: Conceptualization, Data curation, Formal analysis, Investigation, Methodology, Project administration, Resources, Software, Supervision, Validation, Writing – original draft, Writing – review & editing. X-YH: Writing – review & editing.
